# Regression Analysis of ICT Impact Factors on Early Adolescents’ Reading Proficiency in Five High-Performing Countries

**DOI:** 10.3389/fpsyg.2019.01646

**Published:** 2019-07-16

**Authors:** Ya Xiao, Yang Liu, Jie Hu

**Affiliations:** Department of Linguistics and Translation, School of International Studies, Zhejiang University, Hangzhou, China

**Keywords:** ICT impact factors, reading proficiency, multiple linear regression, early adolescent, PISA 2015

## Abstract

The popularity of information and communication technology (ICT) has had a significant influence on the reading proficiency of early adolescents. Achieving excellent reading proficiency, which is related not only to a student’s inherent talent but also to various impact factors, can greatly enhance the effectiveness of reading education. The Program for International Student Assessment (PISA) 2015 provides an international view on the reading proficiency of 15-year-olds in a computer-based testing environment. In this study, a multiple linear regression model was constructed using the computing language R to investigate the association between student-level ICT impact factors (the availability of ICT, the use of ICT and attitudes toward ICT) and reading proficiency among early adolescents. The sample included 37,155 15-year-olds from five representative countries with extremely high reading proficiency. The results showed that the students’ ICT-related attitudinal factors concerning their interest in ICT and perceived autonomy in using ICT, rather than ICT availability and ICT use, were closely associated with high reading proficiency. In addition, ICT devices should be integrated not only as instructional media but also as a cognitive tool for teaching reading with timely and appropriate scrutiny.

## Introduction

The concept of computer-based assessment of reading proficiency is of fundamental significance in the age of information and communication technology (ICT) (Naumann, 2015). The proliferation of ICT has a profound influence on the concept of reading proficiency (e.g., Liu, 2005; Coiro and Dobler, 2007) because it has largely reshaped students’ learning processes and reading activities (e.g., Gan et al., 2015; Mantoro et al., 2017) by engaging students in effective reading activities (e.g., Chen and Hu, 2018) and improving their reading comprehension ability (e.g., Whyte et al., 2014). As the benchmark of international large-scale assessment, the Program for International Student Assessment (PISA) has evaluated reading, science, and mathematics achievement among 15-year-olds from participating countries/economies of the Organization of Economic and Cultural Development (OECD) every 3 years since 2000. Reading proficiency in this influential assessment is recognized as “students’ ability to understand, use, reflect on and engage with written texts in order to achieve one’s goals, develop one’s knowledge and potential, and participate in society” (OECD, 2015, p. 30). This large-scale assessment facilitates the infrastructural and epistemological construction of global education work (Sellar and Lingard, 2013). For the first time, the PISA 2015 delivered the assessments of all three subjects via computer. Among the 72 participating economies, only 15 economies took the paper-based test due to technical problems. These changes have launched a new area of research, that is, the role played by myriad ICT impact factors in students’ reading proficiency because different types of reading activities and related impact factors have emerged (OECD, 2011).

The PISA reading proficiency test has been studied for nearly 20 years. From the long-term perspective, from the PISA 2000 to the PISA 2015, there has been no significant change in the framework of reading assessment among the six consecutive cycles of PISA 2000, PISA 2003, PISA 2006, PISA 2009, PISA 2012, and PISA 2015 (OECD, 2012, 2017). Thus, the whole reading framework and a large number of derived variables in the PISA 2015 were also taken from the previous PISA cycles without change as part of the trend content. In this sixth cycle of PISA assessment, a set of tasks including 103 questions was used in the PISA 2015 reading assessment (OECD, 2016, p. 146). Students’ reading proficiency scores were analyzed based on item response theory and officially released in the *PISA 2015 Results*. The proficiency levels described from the lowest to the highest are Level 1b, Level 1a, Level 2, Level 3, Level 4, Level 5, and Level 6. These seven proficiency levels used in the PISA 2015 reading assessment are the same as those established for the PISA 2009 assessment. The required reading skills at each proficiency level are described according to the three processes by which students answer the questions. These three processes are defined in the framework as “access and retrieve” (skills associated with finding, selecting and collecting information); “integrate and interpret” (processing what is read to make sense of a text); and “reflect and evaluate” (drawing on knowledge, ideas or values external to the text) (OECD, 2016, p. 162).

Starting with the PISA 2009, the OECD, for the first time, designed a computer-based reading assessment as an additional option for its reading proficiency test. Regarding the assessment contents of the paper-based and computer-based PISA 2015 reading proficiency assessment, the latter differs from the former only in format, i.e., the way of presenting long texts by screen and the basic knowledge of hardware usage. However, compared with other cycles of the ICT familiarity questionnaire in the PISA computer-based assessment of reading, four derived variables were newly developed in the PISA 2015, including students’ ICT interest (INTICT), perceived competence in ICT usage (COMPICT), perceived autonomy related to ICT usage (AUTICT) and the degree to which ICT is part of their daily social life (SOIAICT). In particular, the index for ICT use outside of school for academic purposes has changed over time: in the PISA 2006 ICT familiarity questionnaire, this index includes five questions that mainly address students’ degree of using a computer to write papers, create spreadsheets, draw or use graphics programs, use educational software and write computer programs (OECD, 2009). In the PISA 2012, this index is examined using seven measurements of browsing the Internet for schoolwork, using email for communication with other students about schoolwork, using email for communication with teachers and the submission of homework, downloading, uploading or browsing material from the school’s website, checking the school’s website for announcements, doing homework on the computer, and sharing school-related materials with other students (OECD, 2015). Finally, in the PISA 2015, the index is derived from 12 measurements, including all seven measurements that were examined in the PISA 2012. In addition, students’ degrees of browsing the Internet to follow up lessons, using social networks for communication with other students and teachers about schoolwork, doing homework on a mobile device, and downloading learning apps on a mobile device are also included (OECD, 2017).

Substantial effort has been made to investigate the impacts of certain factors on students’ reading proficiency based on the PISA assessment framework. The previous studies can be divided into three categories. The first category is that of sociodemographic factors. Gender, family background and immigration background are confirmed to be significant sociodemographic factors of computer-based assessment measuring reading proficiency. Specifically, 15-year-old girls tend to score higher in computer-based reading assessments on multiple layers of reading skills than boys of the same age (e.g., Stoet and Geary, 2015; Puteh et al., 2016; Torppa et al., 2018). In addition, parental education (e.g., Rajchert et al., 2014), early parental engagement in educational activities (e.g., Hemmerechts et al., 2016), and parental involvement in social and cultural exchange (e.g., Gotoh et al., 2013) are found to be positive factors of reading performance. For immigrant background factors, immigrant students perform consistently worse than native students (e.g., Liberto, 2014), which can be explained by insufficient family support and the control of immigrants (Santos et al., 2016). In the meantime, it has also been found that the sense of school belonging exerts a moderating effect in the mathematical achievement gap between immigrants and natives (Schachner et al., 2017); however, for reading performance, this moderating effect turns out to be insignificant (Mok et al., 2016). The second category is related to cognitive factors. Cognitive skills (rapid naming, phonological awareness, and letter knowledge) and cognitive learning strategies (elaboration and memorization) positively influence reading proficiency (e.g., Li and Chun, 2012; Eklund et al., 2018). The third category concerns instructional factors. Categorical instructions or curricula targeting students of different reading levels improve their reading results (e.g., Shin et al., 2013). In addition, teachers’ guidance of students when they encounter difficulties in reading, teachers’ stimulation of students’ reading processes and the classroom reading environment are all meaningful factors influencing students’ reading proficiency (Meng et al., 2017).

Previous studies have constructed statistical models using the theoretically-based rationale that ICT impact factors are related to reading proficiency. For instance, to examine the mediation effect from individual differences in the inner and outer states of ICT to the PISA reading proficiency, a partial mediation model was constructed (Lee and Wu, 2012). An ordered logit model was employed to estimate relationships between an ordinal dependent variable (i.e., PISA test score) and a set of independent variables (i.e., the student’s background, school characteristics, the home/family environment and the student’s access to ICT facilities) (Erdogdu and Erdogdu, 2015). In these studies, special attention was given to student-level ICT impact factors. Student-level ICT impact factors were obtained from the ICT familiarity questionnaire, which has been gradually developed since the PISA 2000. In the PISA 2015 questionnaire, these factors can be generalized into three main categories: the availability of ICT, the use of ICT and attitudes toward ICT. With regard to the impact of ICT availability, the mere availability of ICT at home is negatively related to reading proficiency, whereas ICT availability at school is not significantly correlated with reading performance (e.g., Lee and Wu, 2012; Hu et al., 2018).

Two relevant contextual factors have been identified in the previous literature with a focus on the impact of ICT use on reading proficiency. The first involves where the ICT is used, i.e., at school or outside of school. The findings regarding ICT use at school are complex; the association between ICT use at school and students’ reading achievement is recognized as having an inverted U-shape, which indicates that overuse of ICT at school may reverse the positive correlation between ICT use at school and students’ reading proficiency (Woessmann and Fuchs, 2005); however, ICT use at school is also found to be negatively correlated with students’ reading proficiency (Petko et al., 2017). Furthermore, this relationship varies among students in different grades. In particular, ICT use at school is found to be positively associated with the reading performance of fourth-grade students whereas it is negatively correlated with that of eighth-grade students (Skryabin et al., 2015). With regard to the second contextual factor, ICT is used outside of school for social entertainment or for web navigation. Specifically, the dimension of social entertainment involves the accessing of email, collaborative gaming, and the use of social media. The dimension of information seeking on the Internet includes reading online news, using e-dictionaries, consulting online encyclopedias and browsing websites for practical information. Some researchers have found that online navigation activities outside of school improve students’ reading proficiency whereas leisure activities decrease it (e.g., Woessmann and Fuchs, 2005; Lee and Wu, 2013). In contrast, some scholars discover that ICT use for entertainment at home is positively correlated with students’ reading performance (Skryabin et al., 2015). Additionally, ICT use for leisure is found to narrow the gender gap in students’ reading scores (e.g., Cheung et al., 2013; Rasmusson and Åberg-Bengtsson, 2015).

Attitude is a significant psychological construct that inheres in or characterizes a person (Richard, 2016). With regard to the ICT attitudinal variables included in the ICT familiarity questionnaire of the PISA 2015, students’ attitudes were found to positively influence students’ reading performance (Lee and Wu, 2012; Petko et al., 2017). In contrast, attitudes toward ICT for social interaction are negatively associated with reading proficiency (Hu et al., 2018). Researchers have used different indexes of ICT attitudes based on the PISA ICT familiarity questionnaire that they selected. For instance, Lee and Wu (2012) obtained one attitudinal index derived from four indicators based on the PISA 2009 ICT familiarity questionnaire. Petko et al. (2017) applied positive attitude toward ICT as a learning tool (ICTATTPOS) derived from six indicators based on the questionnaire in the PISA 2012. Considering that the constructs of ICT attitudes applied in the previous studies are not yet fully developed, a more comprehensive ICT familiarity questionnaire of the PISA 2015 is utilized in the current study to analyze the impacts of students’ ICT-related attitudes; this questionnaire includes four explicit indexes: interest in ICT, perceived ICT competence, perceived autonomy in using ICT, and enjoyment of social communication using ICT (OECD, 2017).

Achieving excellence in education can greatly enhance the effectiveness of education (OECD, 2009); excellence involves more than a student’s inherent talent as it is also related to various interactive factors (Hu and Wei, 2018). Most of the abovementioned studies investigated the ICT impact factors of students’ reading proficiency in one or more countries; however, the literature on the representativeness of countries with excellent reading proficiency remains insufficient. The top-performing countries should receive particular attention since relevant findings would certainly offer innovative insights leading to educational excellence for educators and policymakers around the world (Jerrim, 2015). Certain previous studies have investigated the relationship between impact factors and excellent subject performance by students. For instance, pedagogical impact factors of 4th-grade students with excellent reading proficiency were identified based on Progress in International Reading Literacy Study (PIRLS) (Xiao and Hu, 2019). Regarding the PISA-based analysis, a set of impact factors influencing top students’ science performance was explored (e.g., Chen et al., 2019). However, few of these studies have targeted ICT impact factors and 15-year-olds’ reading proficiency in high-performing countries. Therefore, this study aimed to identify the correlation between ICT impact factors and early adolescents’ reading proficiency in high-performing countries based on the large-scale educational assessment of the PISA 2015. Although the examination of the high-performing countries versus the low-performing ones can maximize the research scope, such comparisons may lead to invalid conclusions and weak representations of educational success because of the polar socioeconomic situations in different countries (OECD, 2016). Therefore, the study’s research objective is to survey the impact of ICT factors on secondary school students’ reading performance in five representative countries with extremely high reading proficiency.

## Materials and Methods

### Sample

The sample was drawn from the PISA 2015 dataset^1^, which is the latest PISA dataset, released in December of 2017. Different from the previous cycles, the assessments of all three domains of science, reading and mathematics were mainly conducted on computers in the PISA 2015. Of the 72 countries/economies that participated in this international assessment, 57 countries/economies (including all 35 OECD members) completed the computer-based assessment (CBA) whereas the remaining 15 participants who lacked computer-test access used the paper-based alternatives. Questionnaires were administered to students, principals, teachers, and parents to obtain relevant contextual information. Only the CBA countries/economies could choose whether to take the ICT familiarity questionnaire (OECD, 2017).

In the case of PISA, students are categorized into seven proficiency levels for each domain based on their test scores: Level 1b is the lowest described level, then Level 1a, Level 2, Level 3 and so on up to Level 6 as the highest proficiency level. Students reaching Level 5 or 6 on the reading proficiency scale are referred to as top performers. Level 6 tasks are more challenging and rigorous than Level 5 tasks. Students reaching Level 6 are typically able to integrate information from multiple texts, understand connotations on a sophisticated level, and expertly handle unfamiliar ideas.

According to the statistical results of the PISA 2015, only countries with at least 2% of performers at Level 6 may be regarded as representative countries with excellent reading proficiency (OECD, 2016) because high-performing educational systems can present better teaching resources, stronger school leadership, higher academic standards, broader educational outcomes, more innovative educational reforms and more international vision than others (Deng and Gopinathan, 2016). Among the seven representative countries with excellent reading proficiency, Canada and Norway did not take the ICT familiarity questionnaire. Thus, in the current study, Singapore (3.600% of Level 6 performers), New Zealand (2.600% of Level 6 performers), Australia (2.000% of Level 6 performers), Finland (2.000% of Level 6 performers) and France (2.000% of Level 6 performers) were selected as the five sample countries across Asia, Europe, and Oceania. Considering the representativeness of these five countries, all students were taken into consideration without distinguishing high- from low-achieving performers. The data of 37,155 sample students were retrieved by Perl computing language version 5.28.2. Boys account for 49.433% of the sample, and girls account for 50.567% of the sample. The age range of the participants was between 15 years and 3 (complete) months and 16 years and 2 (complete) months, as strictly required by the PISA (OECD, 2016, p. 210). In addition, the percentage of individuals with ICT availability at home (ICTHOME) or at school (ICTSCH) is at least 98.640% in five countries, respectively. Students with access to ICT both at home and at school are shown as 99.890% in total. The demographic information is presented in Table 1.

**Table 1:** Demographic information of participants from five representative countries.

Country	Observation	Gender	Participants with ICT availability at school	Participants with ICT availability at home	Participants with ICT availability at school and at home
	
		% female	% yes	% yes	% yes
Australia	14,530	49.298% (7,163/14,530)	99.484% (14,455/14,530)	99.780% (14,498/14,530)	99.876% (14,512/14,530)
Finland	5,882	48.674% (2,863/5,882)	98.640% (5,802/5,882)	99.898% (5,876/5,882)	99.915% (5,877/5,882)
France	6,108	50.933% (3,111/6,108)	98.838% (6,037/6,108)	99.853% (6,099/6,108)	99.935% (6,104/6,108)
New Zealand	4,520	49.934% (2,257/4,520)	99.535% (4,499/4,520)	99.823% (4,512/4,520)	99.978% (4,519/4,520)
Singapore	6,115	48.618% (2,973/6,115)	98.692% (6,035/6,115)	99.920% (6,110/6,115)	99.787% (6,102/6,115)
Total	37,155	49.433% (18,367/37,155)	99.120% (36,828/37,155)	99.812% (37,085/37,155)	99.890% (37,114/37,155)

*Sources: OECD PISA 2015 general database.*

### Data Analysis

#### Variables

As it is impossible for each student to complete all test items, the PISA 2015 computed 10 plausible values (PVs) of reading scores to measure students’ performance (see Table 2). The present study followed the recommendations for addressing PVs in international large-scale assessments (OECD, 2009; Rutkowski et al., 2010), considering all 10 PVs simultaneously as the dependent variables for the purpose of obtaining unbiased and stable estimates.

**Table 2:** Descriptive statistics of plausible values of reading proficiency in the PISA 2015 computer-based reading assessment.

Variable	Description	Mean	*SD*	Minimum	Maximum
PV1READ	Plausible value 1 of reading score	506.691	104.000	73.377	851.085
PV2READ	Plausible value 2 of reading score	507.148	104.337	46.927	844.637
PV3READ	Plausible value 3 of reading score	507.810	104.006	57.679	851.970
PV4READ	Plausible value 4 of reading score	506.642	104.387	83.508	839.131
PV5READ	Plausible value 5 of reading score	507.818	104.958	96.893	865.085
PV6READ	Plausible value 6 of reading score	507.261	103.858	0.000	870.747
PV7READ	Plausible value 7 of reading score	507.667	104.540	28.659	898.018
PV8READ	Plausible value 8 of reading score	507.173	104.202	46.421	849.645
PV9READ	Plausible value 9 of reading score	508.171	104.693	22.847	864.958
PV10READ	Plausible value 10 of reading score	507.018	104.935	81.639	884.906

*Sources: OECD PISA 2015 general database. *N* = 37,155. The dependent variable is students’ reading proficiency, reflected by students’ reading score in the PISA reading test.*

This study included three categories of student-level ICT factors as regressors (see Table 3), i.e., the availability of ICT (at school and outside of school), the use of ICT (at school or outside of school for academic and leisure purposes), and attitudes toward ICT (students’ interest in ICT, perceived autonomy related to ICT, perceived ICT competence, and ICT use for social interaction). In addition, the binary variable Gender and the derived variable of students’ gender and economic, social and cultural status (ESCS) were also considered. Based on the theoretical rationale in this study, all variables related to ICT availability, ICT use and attitudes toward ICT were included in the following analyses.

**Table 3:** Descriptive statistics of ICT availability, ICT use, ICT attitudes and student background based on the PISA 2015 computer-based reading assessment.

Variable	Variable description	Mean	*SD*	Minimum	Maximum
**ICT availability**					
ICTHOME	ICT available at home index	8.600	1.638	0.000	11.000
ICTSCH	ICT available at school index	7.013	1.932	0.000	10.000
**ICT use**					
USESCH	Use of ICT at school in general	0.258	0.826	–1.668	3.629
HOMESCH	ICT use outside of school for schoolwork	–0.058	0.942	–2.691	3.604
ENTUSE	ICT use outside of school leisure	–0.010	0.888	–3.710	4.848
**ICT attitudes**					
INTICT	Students’ ICT interest	0.131	0.935	–2.988	2.819
AUTICT	Students’ perceived autonomy related to ICT use	0.130	0.900	–2.503	2.096
COMPICT	Students’ perceived ICT competence	0.090	0.886	–2.706	2.074
SOIAICT	Students’ ICT as a topic in social interaction	0.095	0.880	–2.136	2.428
**Student background**					
ESCS	Index of economic, social and cultural status	0.107	0.822	–4.692	3.567
Gender	Students’ gender	/	/	0.000	1.000

*Sources: OECD PISA 2015 general database. *N* = 37,155. The independent variables ICTHOME, ICTSCH, USESCH, HOMESCH, ENTUSE, INTICT, AUTICT, COMPICT, SOIAICT and the covariate ESCS were all derived variables based on IRT scaling. Gender is a binary variable, in which female is coded as “0” while male is coded as “1.” The dependent variable is students’ reading proficiency, reflected by students’ reading score in the PISA reading test.*

#### Multiple Linear Regression (MLR) Modeling

A regression model that contains more than one regressor variable is called a multiple regression model (Montgomery and Runger, 2007). An MLR model is “typically employed to measure the effects of the explanatory variables on performance” (Fariña et al., 2015, p. 179). It can accurately reflect the correlations among factors, indicate the degree of fit, and improve the effect of the regression equation (Holmes and Rinaman, 2015). Linear relationships among the various factors can be analyzed intuitively and promptly by using multiple sets of data.

In this study, considering that students’ reading proficiency is associated with multiple factors, it is effective and realistic to estimate the dependent variable by using the optimal combination of multiple independent variables, which can be accurately realized by an MLR model, in line with recommendations for PISA data analysis (Rutkowski et al., 2010). The equation for MLR is

(1) $$\begin{array}{c} y_{i}=\beta_{0}+\beta_{1}x_{i1}+\beta_{2}x_{i2}+...\beta_{p}x_{ip}+\varepsilon \end{array}$$

where

y_i_ refers to the dependent variables,

β_0_ refers to the intercept, and

β_p_ refers to the partial regression coefficient, which gauges the unit change in the dependent variable per unit increase in the factors on the condition that the rest of the factors remain unchanged.

𝜀 refers to the error term.

In the current study, MLR modeling was performed using R computing language version 3.5.0^2^. The data analysis procedure was as follows:

First, the data preprocessing procedure was conducted. Large-scale assessments (e.g., the PISA), conducted in the context of item response theory (Cui et al., 2019), generally contain missing values. In this context, the aggr() function from the R Language package ‘VIM’ was used to visualize the number and proportion of missing values. Deleting the missing values is one solution when the missing rate is lower than 5% for each variable; however, this solution could not be used in this study due to the high missing rate of over 10%. Therefore, to ensure the maximum number of observations, the imputation of missing values was conducted in this study. Many researchers have advocated the use of missForest, a non-parametric method based on the randomForest model, in working with samples that involve different data types (Stekhoven and Bühlmann, 2012; Jin et al., 2015; Finch et al., 2016). Thus, because the sample included in this study contains both continuous and dichotomous variables, the missForest() function was used to impute the missing values.

Second, the correlation coefficients among the nine independent variables and ten PVs of reading performance were computed, and they were within the acceptable limits. Further, the *T*-value and the *F*-value needed to be emphasized to determine the correlation between nine independent variables and reading proficiency.

Third, the lm() function from the core package ‘stats’ was used to compute the MLR model. For each plausible value of reading performance, the model was built by the regressors and covariates.

The summary statistics of variables are presented in Table 3. As there were ten PVs, ten MLR models were eventually produced. The residuals (𝜀), estimates (β), intercept (β_0_), standard error (SE), multiple R-squared (*R*^2^) and *p*-values of the T-statistic and F-statistic are shown in the results for further discussion.

Fourth, assumptions of homoscedasticity and endogeneity were checked. Widely used to verify whether a regression model contains heteroskedastic error (Jeong and Lee, 2008), White’s test (White, 1980) was applied in this study by the computing heteroscedasticity-robust standard error in test statistics (Wooldridge, 2003). Moreover, the problem of endogeneity might exist when the ICT use is an endogenous variable (Fariña et al., 2015). Therefore, the assumption of endogeneity was checked with all three covariates of ICT use, i.e., USESCH, HOMESCH and ENTUSE. The differences between two regression models of with and without any of these covariates for the rest of the variables were calculated and provided in Supplementary Tables S1–S6, respectively. The comparisons of result difference of each ICT use variable were presented in Table 4. No significant differences were found with and without these variables, respectively, in this process.

**Table 4:** Comparison of the results of the regression models with and without each ICT use factor of USESCH, HOMESCH, and ENTUSE.

Factor	Regression model results with USESCH		Regression model results without USESCH		Differences		Regression model results with HOMESCH		Regression model results without HOMESCH		Differences		Regression model results with ENTUSE		Regression model results without ENTUSE		Differences	
ICTHOME	β	–4.331^∗∗∗^ (0.396)	β	–4.438^∗∗∗^ (0.363)	β	0.107 (0.033)	β	–4.331^∗∗∗^ (0.396)	β	–4.334^∗∗∗^ (0.362)	β	0.003 (0.034)	β	–4.331^∗∗∗^ (0.396)	β	–4.775^∗∗∗^ (0.360)	β	0.444 (0.036)
	β^∗^SD	–7.094	β^∗^SD	–7.270	β^∗^SD	0.176	β^∗^SD	–7.094	β^∗^SD	–7.050	β^∗^SD	0.044	β^∗^SD	–7.094	β^∗^SD	–7.821	β^∗^SD	0.727
ICTSCH	β	–3.265^∗∗∗^ (0.295)	β	–3.829^∗∗∗^ (0.288)	β	0.564 (0.007)	β	–3.265^∗∗∗^ (0.295)	β	–3.268^∗∗∗^ (0.294)	β	0.003 (0.001)	β	–3.265^∗∗∗^ (0.295)	β	–3.131^∗∗∗^ (0.296)	β	0.134 (0.001)
	β^∗^SD	–6.308	β^∗^SD	–7.214	β^∗^SD	0.906	β^∗^SD	–6.308	β^∗^SD	–6.314	β^∗^SD	0.006	β^∗^SD	–6.308	β^∗^SD	–6.049	β^∗^SD	0.259
USESCH	β	/	β	/	β	/	β	–7.536^∗∗∗^ (0.779)	β	–7.59^∗∗∗^ (0.710)	β	0.014 (0.069)	β	–7.536^∗∗∗^ (0.779)	β	–8.634^∗∗∗^ (0.780)	β	1.098 (0.001)
	β^∗^SD	/	β^∗^SD	/	β^∗^SD	/	β^∗^SD	–6.225	β^∗^SD	–5.286	β^∗^SD	0.939	β^∗^SD	–6.225	β^∗^SD	–7.132	β^∗^SD	0.907
HOMESCH	β	–0.325^∗∗∗^ (0.700)	β	–3.127^∗∗∗^ (0.640)	β	2.802 (0.060)	β	/	β	/	β	/	β	–0.325^∗∗∗^ (0.700)	β	–8.179^∗∗∗^ (0.728)	β	7.854 (0.028)
	β^∗^SD	–0.306	β^∗^SD	–1.245	β^∗^SD	0.939	β^∗^SD	/	β^∗^SD	/	β^∗^SD	/	β^∗^SD	–7.235	β^∗^SD	–7.263	β^∗^SD	0.028
ENTUSE	β	–8.148^∗∗∗^ (0.746)	β	–9.016^∗∗∗^ (0.745)	β	0.868 (0.001)	β	–8.148^∗∗∗^ (0.746)	β	–8.179^∗∗∗^ (0.728)	β	0.031 (0.042)	β	/	β	/	β	/
	β^∗^SD	–7.236	β^∗^SD	–8.002	β^∗^SD	0.766	β^∗^SD	–7.236	β^∗^SD	–7.263	β^∗^SD	0.027	β^∗^SD	/	β^∗^SD	/	β^∗^SD	/
INTICT	β	9.955^∗∗∗^ (0.661)	β	9.827^∗∗∗^ (0.662)	β	0.128 (0.001)	β	9.955^∗∗∗^ (0.661)	β	9.954^∗∗∗^ (0.661)	β	0.010 (0.00)	β	9.955^∗∗∗^ (0.661)	β	8.513^∗∗∗^ (0.648)	β	1.442 (0.013)
	β^∗^SD	9.308	β^∗^SD	9.190	β^∗^SD	0.118	β^∗^SD	9.308	β^∗^SD	9.307	β^∗^SD	0.001	β^∗^SD	9.308	β^∗^SD	8.359	β^∗^SD	0.949
AUTICT	β	23.529^∗∗∗^ (0.775)	β	23.673^∗∗∗^ (0.776)	β	0.144 (0.001)	β	23.529^∗∗∗^ (0.775)	β	23.533^∗∗∗^ (0.774)	β	0.004 (0.001)	β	23.529^∗∗∗^ (0.775)	β	23.630^∗∗∗^ (0.772)	β	0.101 (0.003)
	β^∗^SD	21.076	β^∗^SD	21.464	β^∗^SD	0.388	β^∗^SD	21.076	β^∗^SD	21.180	β^∗^SD	0.104	β^∗^SD	21.076	β^∗^SD	20.367	β^∗^SD	0.709
COMPICT	β	–2.931^∗∗∗^ (0.796)	β	–3.206^∗∗∗^ (0.794)	β	0.275 (0.002)	β	–2.931^∗∗∗^ (0.796)	β	–2.934^∗∗∗^ (0.794)	β	0.003 (0.002)	β	–2.931^∗∗∗^ (0.796)	β	–3.208^∗∗∗^ (0.787)	β	0.277 (0.009)
	β^∗^SD	–2.597	β^∗^SD	–2.844	β^∗^SD	0.247	β^∗^SD	–2.597	β^∗^SD	–2.600	β^∗^SD	0.003	β^∗^SD	–2.497	β^∗^SD	–1.559	β^∗^SD	0.938
SOIAICT	β	–16.001^∗∗∗^ (0.709)	β	–16.321^∗∗∗^ (0.710)	β	0.320 (0.001)	β	–16.001^∗∗∗^ (0.709)	β	–16.014^∗∗∗^ (0.705)	β	0.013 (0.004)	β	–16.001^∗∗∗^ (0.709)	β	–16.937^∗∗∗^ (0.705)	β	0.936 (0.004)
	β^∗^SD	–14.065	β^∗^SD	–14.351	β^∗^SD	0.286	β^∗^SD	–14.065	β^∗^SD	–14.076	β^∗^SD	0.011	β^∗^SD	–14.065	β^∗^SD	–14.887	β^∗^SD	0.822
ESCS	β	47.930^∗∗∗^ (0.663)	β	–47.644^∗∗∗^ (0.665)	β	0.286 (0.002)	β	47.930^∗∗∗^ (0.663)	β	47.920^∗∗∗^ (0.660)	β	0.010 (0.003)	β	47.930^∗∗∗^ (0.663)	β	48.320^∗∗∗^ (0.664)	β	0.390 (0.001)
	β^∗^SD	39.398	β^∗^SD	39.146	β^∗^SD	0.252	β^∗^SD	39.398	β^∗^SD	39.396	β^∗^SD	0.002	β^∗^SD	39.398	β^∗^SD	39.719	β^∗^SD	0.321
Gender (female = 0)	β	–28.506^∗∗∗^ (1.039)	β	–28.363^∗∗∗^ (1.040)	β	0.143 (0.001)	β	–28.506^∗∗∗^ (1.039)	β	–28.488^∗∗∗^ (1.035)	β	0.018 (0.004)	β	–28.506^∗∗∗^ (1.039)	β	–29.966^∗∗∗^ (0.826)	β	1.460 (0.213)
	β^∗^SD	–14.253	β^∗^SD	–14.181	β^∗^SD	0.072	β^∗^SD	–14.253	β^∗^SD	–14.244	β^∗^SD	0.009	β^∗^SD	–14.253	β^∗^SD	–14.983	β^∗^SD	0.730

*The coefficient of the regression model presented in this table were the mean coefficient of the 10 models. Heteroscedasticity-robust standard errors are listed in parentheses. Results of the model with and without USESCH, HOMESCH and ENTUSE were compared in Supplementary Tables S2, S4, S6, respectively, with the main indicator of β^∗^SD. Significant codes: ^∗∗∗^*p* < 0.05.*

## Results

This article aimed to examine the influence of ICT impactors on students’ reading proficiency in high-achieving countries; therefore, the five representative countries were assessed as a cohort with high-achieving reading proficiency.

### Demographic Covariates

Regarding the PISA reading proficiency, the fundamental demographic factors involved the ESCS and gender (Petko et al., 2017; Hu et al., 2018). Thus, these two factors were included as the two demographic covariates in this study. Both ESCS (β = 47.930, *SE* = 0.663, *p* < 0.001) and gender (β = -28.506, *SE* = 1.039, *p* < 0.010) were significantly correlated with reading proficiency in Table 5. Specifically, ESCS was positively associated with the students’ reading performance. For a one-point increment in ESCS, the students’ reading scores increased by 39.398 points (β^∗^SD), which demonstrated that the students in countries with higher ESCS tended to achieve better reading results.

**Table 5:** The effect of ICT impact factors on reading proficiency.

Factor	Model 1 PV1READ		Model 2 PV2READ		Model 3 PV3READ		Model 4 PV4READ		Model 5 PV5READ		Model 6 PV6READ		Model 7 PV7READ		Model 8 PV8READ		Model 9 PV9READ		Model 10 PV10READ		Mean	
	**β**	**β^∗^SD**	**β**	**β^∗^SD**	**β**	**β^∗^SD**	**β**	**β^∗^SD**	**β**	**β^∗^SD**	**β**	**β^∗^SD**	**β**	**β^∗^SD**	**β**	**β^∗^SD**	**β**	**β^∗^SD**	**β**	**β^∗^SD**	**β**	**β^∗^SD**
**ICT availability**				
ICTHOME	–4.434^∗∗∗^ (0.700)	–7.263	–4.162^∗∗∗^ (0.362)	0.333	–4.721^∗∗∗^ (0.358)	0.331	–3.937^∗∗∗^ (0.361)	0.332	–4.209^∗∗∗^ (0.368)	0.335	–3.869^∗∗∗^ (0.361)	0.330	–4.383^∗∗∗^ (0.364)	0.333	–4.306^∗∗∗^ (0.360)	0.332	–4.456^∗∗∗^ (0.363)	0.334	–4.832^∗∗∗^ (0.362)	0.334	–4.331^∗∗∗^ (0.396)	–7.094
ICTSCH	–3.243^∗∗∗^ (0.293)	–6.265	–3.313^∗∗∗^ (0.295)	0.271	–2.972^∗∗∗^ (0.292)	0.270	–3.131^∗∗∗^ (0.296)	0.271	–3.047^∗∗∗^ (0.296)	0.274	–3.493^∗∗∗^ (0.291)	0.270	–3.535^∗∗∗^ (0.297)	0.232	–3.684^∗∗∗^ (0.294)	0.271	–3.030^∗∗∗^ (0.295)	0.273	–3.201^∗∗∗^ (0.298)	0.273	–3.265^∗∗∗^ (0.295)	–6.308
**ICT use**																						
USESCH	–7.20^∗∗∗^ (0.782)	–5.947	–7.987^∗∗∗^ (0.781)	0.674	7.275^∗∗∗^ (0.764)	0.671	–8.042^∗∗∗^ (0.783)	0.673	–6.425^∗∗∗^ (0.784)	0.680	–7.673^∗∗∗^ (0.774)	0.670	–7.394^∗∗∗^ (0.780)	0.744	–7.807^∗∗∗^ (0.782)	0.742	–7.489^∗∗∗^ (0.776)	0.747	–8.070^∗∗∗^ (0.787)	0.677	–7.536^∗∗∗^ (0.779)	–6.225
HOMESCH	–0.246 (0.700)	–0.232	–0.464 (0.702)	0.660	–0.574 (0.964)	0.657	–0.193 (0.695)	0.658	0.300 (0.705)	0.665	–0.144 (0.695)	0.655	–0.989 (0.696)	0.660	0.994 (0.705)	0.657	–1.272 (0.702)	0.662	–0.663 (0.708)	0.663	–0.325 (0.700)	–0.306
ENTUSE	–8.884^∗∗∗^ (0.746)	–7.89	–8.102^∗∗∗^ (0.752)	0.662	–8.190^∗∗∗^ (0.741)	0.660	–7.654^∗∗∗^ (0.736)	0.661	–8.083^∗∗∗^ (0.7540	0.667	–7.973^∗∗∗^ (0.745)	0.658	–7.768^∗∗∗^ (0.740)	0.680	–8.285^∗∗∗^ (0.759)	0.678	–8.002^∗∗∗^ (0.739)	0.683	–8.541^∗∗∗^ (0.752)	0.665	–8.148^∗∗∗^ (0.746)	–7.236
**ICT attitudes**				
INTICT	9.821^∗∗∗^ (0.662)	9.183	9.376^∗∗∗^ (0.666)	0.637	10.249^∗∗∗^ (0.658)	0.635	9.630^∗∗∗^ (0.657)	0.636	9.787^∗∗∗^ (0.663)	0.643	10.084^∗∗∗^ (0.654)	0.633	9.821^∗∗∗^ (0.665)	0.637	10.519^∗∗∗^ (0.660)	0.636	10.017^∗∗∗^ (0.664)	0.630	10.242^∗∗∗^ (0.658)	0.640	9.955^∗∗∗^ (0.661)	9.308
AUTICT	23.055^∗∗∗^ (0.767)	20.795	24.482^∗∗∗^ (0.778)	0.750	22.940^∗∗∗^ (0.770)	0.747	23.826^∗∗∗^ (0.771)	0.748	23.504^∗∗∗^ (0.782)	0.756	22.997^∗∗∗^ (0.770)	0.745	23.286^∗∗∗^ (0.778)	0.750	23.903^∗∗∗^ (0.776)	0.748	23.221^∗∗∗^ (0.773)	0.753	24.075^∗∗∗^ (0.780)	0.753	23.529^∗∗∗^ (0.775)	21.076
COMPICT	–2.874^∗∗∗^ (0.790)	–2.546	–3.015^∗∗∗^ (0.804)	0.766	–2.238^∗∗∗^ (0.790)	0.763	–3.243^∗∗∗^ (0.7880	0.764	–3.043^∗∗∗^ (0.799)	0.772	–2.404^∗∗∗^ (0.792)-	0.761	–1.831^∗∗∗^ (0.800)	0.765	–3.986^∗∗∗^ (0.795)	0.763	–3.105^∗∗∗^ (0.794)	0.768	–3.572^∗∗∗^ (0.792)	0.769	–2.931^∗∗∗^ (0.796)	–2.597
SOIAICT	–15.693^∗∗∗^ (0.709)	–13.794	–16.337^∗∗∗^ (0.711)	0.687	–16.752^∗∗∗^ (0.703)	0.685	–16.192^∗∗∗^ (0.705)	0.684	–16.055^∗∗∗^ (0.717)	0.693	–15.743^∗∗∗^ (0.700)	0.682	–15.895^∗∗∗^ (0.711)	0.690	–16.064^∗∗∗^ (0.713)	0.688	–15.525^∗∗∗^ (0.711)	0.693	–15.755^∗∗∗^ (0.713)	0.690	–16.001^∗∗∗^ (0.709)	–14.065
**Student background**				
ESCS	48.210^∗∗∗^ (0.656)	39.629	47.380^∗∗∗^ (0.667)	0.631	47.676^∗∗∗^ (0.660)	0.628	48.116^∗∗∗^ (0.664)	0.630	47.640^∗∗∗^ (0.673)	0.636	47.767^∗∗∗^ (0.656)	0.626	47.884^∗∗∗^ (0.666)	0.632	47.885^∗∗∗^ (0.663)	0.630	48.188^∗∗∗^ (0.661)	0.635	48.549^∗∗∗^ (0.665)	0.633	47.930^∗∗∗^ (0.663)	39.398
Gender (female = 0)	–28.410^∗∗∗^ (1.035)	–14.205	–27.647^∗∗∗^ (1.040)	1.020	–27.441^∗∗∗^ (1.037)	1.017	–29.484^∗∗∗^ (1.037)	1.018	–28.844^∗∗∗^ (1.047)	1.029	–28.933^∗∗∗^ (1.032)	1.013	–30.656^∗∗∗^ (1.039)	1.023	–27.921^∗∗∗^ (1.035)	1.021	–28.661^∗∗∗^ (1.043)	1.028	–27.062^∗∗∗^ (1.044)	1.025	–28.506^∗∗∗^ (1.039)	–14.253

*N = 37,155. The dependent variable is students’ readings score. Models 1 to 10 refer to the regression models for ten plausible values of reading score. In the PISA 2015, each student has 10 plausible values of reading scores (PV1READ∼PV10READ). A higher plausible value reflects a higher reading proficiency. The regression model is estimated using Equation. Since the independent variables were derived based on IRT scaling, with one percent change in the independent variable, the dependent variable is changed by the coefficient multiplied by its standard deviation (β^∗^SD). Heteroscedasticity-robust standard errors are listed in parentheses. Significant codes: ^∗∗∗^*p* < 0.001.*

Table 5 presents the results for all required coefficients for the statistically significantly related factors included in the optimal MLR model. As shown, the explained variance for the model varied from *R*^2^ = 0.209 to *R*^2^ = 0.214. In the fields of humanities and social sciences, these *R*^2^ values were within an acceptable range because it was not expected that all relevant variables would be included to indicate the subjects’ behavior. In the existing studies of regression analysis using the PISA dataset (e.g., Chiacchio et al., 2016; Naumann and Sälzer, 2017; Tay et al., 2017), the maximum *R*^2^ reached 0.310, 0.239, and 0.230, respectively. Even if the *R*^2^ was low in this study, the factors were significantly correlated, which means that important conclusions could still be drawn from the model (Neter et al., 2012). The detailed information of all statistical analyses conducted in this study are available upon request.

### ICT-Related Factors

As shown in Table 5, ICT availability at home (β = -4.331, *SE* = 0.396, *p* < 0.001) and at school (β = -3.265, *SE* = 0.295, *p* < 0.001) was negatively associated with students’ reading proficiency: with a one-point improvement in the availability of ICT at home and at school, students’ reading scores decreased by -7.094 and -6.308 points (β^∗^SD), respectively. Regarding use, ICT use at school in general (β = -7.536, *SE* = 0.779, *p* < 0.001) was negatively related to reading performance; reading scores were decreased by 6.225 points (β^∗^SD), with one-point growth in the use of ICT at school. The use of ICT outside of school for entertainment (β = -8.148, *SE* = 0.746, *p* < 0.001) indicated a negative correlation with reading proficiency; the use of ICT outside of school for entertainment was increased by one point, and reading scores dropped by 7.236 points (β^∗^SD). No significant association was found between the use of ICT outside of school for schoolwork and reading proficiency. With regard to students’ attitudes toward ICT, all attitudinal factors examined were significantly related to reading performance: interest in ICT (β = 9.955, *SE* = 0.661, *p* < 0.001) and perceived autonomy related to ICT use (β = 23.529, *SE* = 0.775, *p* < 0.001) were positively related to reading scores, whereas perceived ICT competence (β = -2.931, *SE* = 0.796, *p* < 0.001) and enjoyment of social interactions through ICT (β = -16.001, *SE* = 0.709, *p* < 0.001) were negatively associated with reading performance. Specifically, reading scores increased by 9.308 and 21.076 (β^∗^SD) points with every one-point increase in students’ interest in ICT and perceived ICT autonomy, respectively. Conversely, with a one-point improvement in perceived ICT competence, students’ reading score decreased by 2.597 points (β^∗^SD). One point of growth in their enjoyment of ICT use for social interaction was found to reduce reading scores by 14.065 points (β^∗^SD).

Moreover, the relationship between ICT impact factors and students’ reading proficiency in each of the five performing countries was also investigated through the same procedure, respectively, in Table 6. As shown, the ICT availability at home (ICTHOME) and the gender (Gender) remained negatively associated with students’ reading proficiency in each of the five counties, and the interest in ICT (INTICT) and the ESCS remained positively correlated with students’ reading proficiency in each of the five counties. For the remaining factors, they were differently associated with reading proficiency among different countries. These results indicated that: four factors (i.e., ICTHOME, INTICT, ESCS, and Gender) were simultaneously identified for all five countries as closely relevant to the students’ reading proficiency, whereas the other factors were differently associated with students’ reading proficiency among countries. For example, the ICT availability at school (ICTSCH) was negatively associated with reading proficiency in Australia (*p* = 0.021, β = -1.296, *SE* = 0.562), France (*p* < 0.001, β = -9.138, *SE* = 0.725) and New Zealand (*p* < 0.001, β = -3.478, *SE* = 0.978). The correlation was insignificant in Finland (*p* = 0.098) and Singapore (*p* = 0.068).

**Table 6:** The effect of ICT impact factors on reading proficiency in each of the five countries.

Group of factors	Measurement	Australia	Finland	France	New Zealand	Singapore	All five countries
**ICT availability**				
ICTHOME	*p*-value	0.000	0.000	0.002	0.001	0.000	0.000
	β	–2.307	–6.68	–3.163	–3.425	–3.353	–4.331
	Robust SE	0.611	0.859	1.001	1.053	0.717	0.396
	β^∗^SD	–3.576	–10.354	–4.903	–5.309	–5.197	–7.094
ICTSCH	*p*-value	0.021	**0**.**098**	0.000	0.000	**0**.**068**	0.000
	β	–1.296	1.008	–9.138	–3.478	–1.091	–3.265
	Robust SE	0.562	0.609	0.725	0.978	0.598	0.295
	β^∗^SD	0.034	1.635	–14.822	–5.641	–1.770	–6.308
**ICT use**				
USESCH	*p*-value	0.000	0.000	**0**.**275**	0.000	0.000	0.000
	β	5.818	–15.801	1.768	–16.562	–8.532	–7.536
	Robust SE	1.577	1.938	1.619	2.685	1.561	0.779
	β^∗^SD	4.311	–11.709	1.310	–12.272	–6.322	–6.225
HOMESCH	*p*-value	**0**.**866**	0.010	0.001	0.008	0.000	0.468
	β	0.213	–3.870	–5.651	6.207	6.432	–0.325
	Robust SE	1.262	1.505	1.647	2.337	1.719	0.700
	β^∗^SD	0.193	–3.510	–5.125	5.630	5.834	–0.306
ENTUSE	*p*-value	0.000	**0**.**226**	0.000	0.000	**0**.**072**	0.000
	β	–18.430	–2.003	–5.965	–8.780	–3.418	–8.148
	Robust SE	1.289	1.655	1.657	2.294	1.901	0.746
	β^∗^SD	–16.163	–1.757	–5.231	–7.700	–2.998	–7.236
**ICT attitudes**				
INTICT	*p*-value	0.000	0.000	0.000	0.000	0.000	0.000
	β	14.716	7.875	0.173	12.993	8.274	9.955
	Robust SE	1.182	1.617	1.485	1.967	1.425	0.661
	β^∗^SD	13.038	6.977	0.153	11.512	7.331	9.308
AUTICT	*p*-value	0.000	0.000	**0**.**907**	0.000	0.000	0.000
	β	22.733	15.973	20.775	18.673	22.32	23.529
	Robust SE	1.350	1.662	1.941	2.194	1.573	0.775
	β^∗^SD	20.028	14.072	18.303	16.451	19.664	21.076
COMPICT	*p*-value	**0**.**616**	**0**.**461**	**0**.**195**	**0**.**202**	0.001	0.003
	β	0.703	–1.311	2.251	2.967	–6.138	–2.931
	Robust SE	1.403	1.779	1.735	2.326	1.802	0.796
	β^∗^SD	0.595	–1.109	1.904	2.510	–5.193	–2.597
SOCIAICT	*p*-value	0.000	**0**.**965**	0.000	0.000	0.000	0.000
	β	–22.012	–0.070	–14.048	–23.675	–21.634	–16.001
	Robust SE	1.270	1.599	1.556	2.208	1.526	0.709
	β^∗^SD	–18.204	–0.058	–11.618	–19.579	–17.891	–14.065
**Student background**				
ESCS	*p*-value	0.000	0.000	0.000	0.000	0.000	0.000
	β	43.679	40.262	57.960	49.631	45.237	48.549
	Robust SE	1.112	1.580	1.667	1.990	1.396	0.665
	β^∗^SD	35.293	32.532	46.832	40.102	36.551	39.398
Gender	*p*-value	0.000	0.000	0.000	0.000	0.000	0.000
	β	–26.038	–46.999	–22.714	–31.721	–14.157	–28.506
	Robust SE	1.696	2.348	2.589	3.032	2.338	1.039
	β^∗^SD	–13.019	–23.500	–11.357	–15.861	–7.079	–14.253

*Coefficients that resulted significant considering a 0.05 significance level appear in bold. Heteroscedasticity-robust standard error was computed to test the potential heteroscedasticity.*

## Discussion

### The Availability of ICT

The availability of ICT includes ICT availability at home (ICTHOME) and ICT availability at school (ICTSCH) (OECD, 2016). On one hand, ICTHOME is found to be inversely related to students’ reading achievement in high-achieving countries, which is consistent with the previous research (Lee and Wu, 2013). This finding might be explained by the low quality of students’ ICT use at home without proper guidance and timely supervision from their parents. Students with access to ICT devices at home (e.g., computers, cell phones, e-books, printers, portable music players) do possess more computer skills (Kuhlemeier and Hemker, 2007) and tend to perform better on reading when assessed by computer (Rasmusson and Åberg-Bengtsson, 2015). However, the overuse or abuse of ICT tends to form detrimental habits such as addiction to computer games, which in turn lowers reading proficiency (Rasmusson and Åberg-Bengtsson, 2015). Hence, parents are suggested to carefully monitor their children’s access to ICT facilities at home and to appropriately direct them to utilize online resources in a reasonable way (Lee and Wu, 2012). On the other hand, ICTSCH is negatively correlated with students’ reading performance in this study, which is consistent with Lai’s (2016) study. This result is closely related to ICT use at school, which is discussed in detail in the next section.

### The Use of ICT

The use of ICT contains ICT use at school in general (USESCH), ICT use at home for schoolwork (HOMESCH), and ICT use at home for leisure (ENTUSE) (OECD, 2016). ICT use at school is negatively related to students’ reading scores, which is consistent with the findings of previous studies (Petko et al., 2017; Tay et al., 2017; Hu et al., 2018). During the process of using ICT in everyday education, teachers may encounter a number of barriers. Ertmer (1999) classified these barriers into two categories: extrinsic and intrinsic barriers. Extrinsic barriers include lack of access, time, support, resources and training, and intrinsic barriers include attitudes, beliefs, practices and resistance. In terms of intrinsic barriers with regard to teachers’ preparedness and perception, although teachers believe ICT use in education is beneficial and may be able to adeptly use the Internet, e-mail, Microsoft Word and PowerPoint for reading teaching, they might possess only limited knowledge in using ICT for more advanced functions, e.g., spreadsheets, concept mapping, programing languages, multimedia authoring and modeling software to compose adapted teaching materials or tailored approaches for students with different reading levels. This indicates a situation where the use of ICT in class is restricted to basic pedagogical practices rather than being effectively integrated into the school curriculum (Aydin, 2013). Therefore, schools are supposed to organize training programs to equip teachers with important ICT knowledge and sufficient ICT skills as well as provide in-time technical support once teachers encounter any difficulty in using ICT in class and so forth (Hadi and Zeinab, 2012). In this case, teachers would be able to use ICT as cognitive tools in class, contributing to an ideal technology-assisted learning environment (e.g., Kommers et al., 2001; Nissen and Tea, 2012; Wei and Hu, 2018; Wei et al., 2018).

The results regarding the influence of the use of ICT for academic purposes outside of school on reading proficiency have varied across the previous studies. In this study, no significant connection is found between ICT use at home for schoolwork and reading proficiency. In the existing studies, Petko et al. (2017) discovered that ICT use for schoolwork outside of school is positively associated with students’ reading performance, which aligns with the research finding of Skryabin et al. (2015). In contrast, Gumus and Atalmis (2011) discovered the negative relationship of ICT academic use at home. These conflicting results might be explained by the fact that the PISA ICT questionnaire have changed over time, as explained in the introduction. In detail, Skryabin et al. (2015) and Petko et al. (2017) applied the ICT questionnaire in the PISA 2012, at which time the index for ICT use outside of school for academic purposes was determined by seven measurements. In Gumus and Atalmis (2011) study, this index was based on five questions in the PISA 2006 ICT questionnaire (OECD, 2006). However, in the current study, the final index of ICT use outside of school for schoolwork is derived from twelve indexes in the PISA 2015 ICT questionnaire, including all seven indexes that were examined in the PISA 2012 (OECD, 2017).

In this study, ICT use outside of school for entertainment is found to be inversely correlated with reading proficiency, which contradicts the findings of some of the past studies. For instance, Gumus and Atalmis (2011) proposed that using ICT devices for leisure, such as playing computer games, may alleviate Turkish students’ stress, increase their momentum, and inspire them to learn more efficiently. However, the pattern of a negative correlation between ICT use outside of school for entertainment and reading performance is found in high-achieving countries (Woessmann and Fuchs, 2005; OECD, 2006, 2015; Petko et al., 2017). Another possible explanation might be the opportunity cost of spending most of the time online outside school for entertainment rather than spending that time reading (Petko et al., 2017). Educators should devote more effort to monitoring and evaluating students’ reading strategies to achieve meaningful e-teaching outcomes. However, ICT use at school in Australia is positively correlated with students’ reading proficiency, which might be caused by the education policies in Australia. These policies contribute a lot to the effectively use of ICT in schools (Radhika and Wu, 2015).

### Attitudes Toward ICT

Regarding attitudes toward ICT, two attitudinal factors, i.e., students’ interest and perceived autonomy in using ICT, are closely associated with the reading proficiency in high-performing countries in this study. This finding is novel, as few studies have confirmed the predominant significant role of ICT-related motivation and self-efficacy in reading scores beyond students’ capabilities. The previous studies have found that the impact of these two attitudinal factors on students’ achievement scores is complex (e.g., Papanastasiou et al., 2004; Lee and Wu, 2012). Lee and Wu (2012) observed that students’ perceptions of educational technology were positively correlated with their academic performance based on the PISA 2009 dataset whereas Papanastasiou et al. (2004) suggested a negative correlation. The reason for the fundamental influence of interest might be the digital learning potential reflected by the items measuring students’ interest in ICT in the PISA 2015 ICT familiarity questionnaire. This potential is measured by two main items: (1) The Internet is a great resource for obtaining information in which I am interested, and (2) I am really excited about discovering new digital devices or applications (OECD, 2017). In effect, these two questions reflect students’ acceptance of ICT related technology. ICT has brought tremendous change by offering readers the opportunity to engage in more flexible reading activities via computers. Nonetheless, many adolescents born in the 1990s have uninterested, skeptical or even fearful attitudes toward e-learning because of the complicated and misleading navigation, non-intuitive design, and user-unfriendly operations, which might hinder their access to informative resources (Hyman et al., 2014). This attitude of rejection decreases students’ autonomy in utilizing ICT facilities for learning. Students without an interest in applying ICT to help them with their work are unlikely to delve into the manuals of electronic devices, choose helpful applications or install updated learning software independently. Hence, students’ indifference to ICT shows little possibility for automatic e-learning in further study, which may hinder their reading performance. This interpretation seems plausible in light of the previous studies on the gender gap in online reading, which observe that the advantage in female students over their male counterparts in paper-based reading decreases when they read online. Based on Bandura’s self-efficacy theory (1993), it is possible that boys’ greater interest and girls’ higher anxiety in the electronic reading environment contributed to the smaller gender gap in digital reading (Nele and Franziska, 2019). Therefore, new effective and technological tools used in the classroom should be geared to students’ interest; in particular, attractive educational applications could trigger students’ positive attitudes or behavior in class (Mera et al., 2019).

With regard to students’ perceived ICT competence in using digital devices, this study finds a slightly negative association. In the meantime, students’ ICT use for social interaction is negatively correlated with reading proficiency in the sample countries. In the PISA 2015 ICT familiarity questionnaire, the questions on this index can be generalized into two categories. One category is ICT as a theme of social communication, and the other is ICT use for social interaction. Although students might receive assistance in using digital devices from social media, using ICT for social communication exerts a greater negative correlation with reading proficiency. This result is consistent with those of previous studies (e.g., Fox et al., 2009; Jacobsen and Forste, 2011) that confirmed that concurrent ICT use for social communication and for reading were negatively associated with their efficiency. Jacobsen and Forste (2011) further proposed that the metacognitive mechanism behind this negative correlation is the distraction of attention and the impairment of short-term memory when performing multiple tasks. Additionally, this finding further explains the negative impact of ICT use at school as mentioned above. Areepattamannil and Khine (2017) revealed the close connection between the frequency of ICT use for social interaction and ICT use at school. In this case, the fact that ICT’s use at school is negatively associated with reading scores is attributed not only to teachers’ behavior but also to students’ reading activities at school. To solve this problem, appropriate direction and timely scrutiny are necessary to prevent students from becoming obsessed with online entertainment such as playing computer games and engaging in social networking activities. The significant negative impact of social interaction activities on students’ reading proficiency in high-achieving countries reflects the fact that social media addiction poses a great threat to reading proficiency with the popularity of ICT.

## Conclusion

This study used multiple linear regression models to analyze the relationship between ICT impact factors and early adolescents’ reading proficiency in five countries with extremely high reading proficiency. It was found that students’ attitudes toward ICT including interest levels and perceived autonomy contributed most to students’ high reading proficiency, rather than ICT availability or ICT use. The current study makes the following three primary contributions to the field: (a) This study delves into the association between the proposed ICT-related factors and students’ reading proficiency in the context of representative countries with excellent reading proficiency based on the latest PISA dataset, and it makes reasonable inferences for illustration; (b) The study reflects upon the application of educational technology in an ICT-assisted learning environment and gives constructive advice with regard to the findings; and (c) Based on the previous literature, this study offers a comprehensive overview of how ICT influences reading performance.

Future research should address a few suggestions. First, considering the exploratory nature of the research, if possible, in the future, longitudinal research can expand the scale of the research. Second, since most of the questions in the PISA questionnaire were the self-reported answers of students, the endogeneity of variables might be a problem. In a pioneering PISA study (Fariña et al., 2015), the Hausman test (Hausman, 1978) was used to diagnose the appropriateness of the endogeneity assumption. Furthermore, propensity score matching approach can be applied to avoid the self-selection problem and obtain an unbiased sample (e.g., Crespo-Cebada et al., 2014). Although this problem does not exist in this study, it still deserves special attention in future research. Additionally, the application of more advanced statistical model, for instance, a linear mixed-effects model (e.g., Hesselmann, 2018), is also essential for future PISA-based studies.

## Data Availability

The data that support the findings of this study are available at http://www.oecd.org/pisa/data/. This is public data released by the OECD.

## Ethics Statement

This study was approved by the Research Ethics Board of Zhejiang University and granting agency, and was performed in accordance with the relevant guidelines and regulations.

## Author Contributions

YX designed the study, analyzed and interpreted the data, and wrote and revised the manuscript. YL revised the manuscript. JH supervised the study, designed the study, interpreted the data, and wrote and revised the manuscript.

## Conflict of Interest Statement

The authors declare that the research was conducted in the absence of any commercial or financial relationships that could be construed as a potential conflict of interest.

## Footnotes

1 http://www.oecd.org/pisa/data/2015database/

2 https://www.r-project.org/
