# Supplementary material for: Regression Analysis of ICT Impact Factors on Early Adolescents’ Reading Proficiency in Five High-Performing Countries
Source: Front Psychol. 2019 Jul 16;10:1646. doi: 10.3389/fpsyg.2019.01646 (PMC6646718; doi:10.3389/fpsyg.2019.01646)
Supplement: Supplementary file 2 [file Table_2.docx]

# Supplementary Table S2. Comparison of the results of the regression models with and without USESCH.

| Factor | Regression model results with USESCH | | Regression model results without USESCH | | Differences | |
| --- | --- | --- | --- | --- | --- | --- |
| ICTHOME | β | -4.331***  (0.396) | β | -4.438***  (0.363) | β | 0.107  (0.033) |
|  | β*SD | -7.094 | β*SD | -7.270 | β*SD | 0.176 |
| ICTSCH | β | -3.265***  (0.295) | β | -3.829***  (0.288) | β | 0.564  (0.007) |
|  | β*SD | -6.308 | β*SD | -7.214 | β*SD | 0.906 |
| HOMESCH | β | -0.325***  (0.700) | β | -3.127***  (0.640) | β | 2.802  (0.060) |
|  | β*SD | -0.306 | β*SD | -1.245 | β*SD | 0.939 |
| ENTUSE | β | -8.148***  (0.746) | β | -9.016***  (0.745) | β | 0.868  (0.001) |
|  | β*SD | -7.236 | β*SD | -8.002 | β*SD | 0.766 |
| INTICT | β | 9.955***  (0.661) | β | 9.827***  (0.662) | β | 0.128  (0.001) |
|  | β*SD | 9.308 | β*SD | 9.190 | β*SD | 0.118 |
| AUTICT | β | 23.529***  (0.775) | β | 23.673***  (0.776) | β | 0.144  (0.001) |
|  | β*SD | 21.076 | β*SD | 21.464 | β*SD | 0.388 |
| COMPICT | β | -2.931***  (0.796) | β | -3.206***  (0.794) | β | 0.275  (0.002) |
|  | β*SD | -2.597 | β*SD | -2.844 | β*SD | 0.247 |
| SOIAICT | β | -16.001***  (0.709) | β | -16.321***  (0.710) | β | 0.320  (0.001) |
|  | β*SD | -14.065 | β*SD | -14.351 | β*SD | 0.286 |
| ESCS | β | 47.930***  (0.663) | β | 47.644***  (0.665) | β | 0.286  (0.002) |
|  | β*SD | 39.398 | β*SD | 39.146 | β*SD | 0.252 |
| Gender (female = 0) | β | -28.506***  (1.039) | β | -28.363***  (1.040) | β | 0.143  (0.001) |
|  | β*SD | -14.253 | β*SD | -14.181 | β*SD | 0.072 |

Note. The coefficient of the regression model presented in this table were the mean coefficient of the 10 models. Heteroscedasticity-robust standard errors are listed in parentheses. The result of the model with USESCH (see Table 5) and that without USESCH (see Supplementary Table S1) were compared and no significant difference was found (see Supplementary Table S2).
