# Supplementary material for: Regression Analysis of ICT Impact Factors on Early Adolescents’ Reading Proficiency in Five High-Performing Countries
Source: Front Psychol. 2019 Jul 16;10:1646. doi: 10.3389/fpsyg.2019.01646 (PMC6646718; doi:10.3389/fpsyg.2019.01646)
Supplement: Supplementary file 4 [file Table_4.docx]

# Supplementary Table S4. Comparison of the results of the regression models with and without HOMESCH.

| Factor | Regression model results with HOMESCH | | Regression model results without HOMESCH | | Differences | |
| --- | --- | --- | --- | --- | --- | --- |
| ICTHOME | β | -4.331^***^ (0.396) | β | -4.334^***^ (0.362) | β | 0.003 (0.034) |
|  | β*SD | -7.094 | β*SD | -7.050 | β*SD | 0.044 |
| ICTSCH | β | -3.265^***^ (0.295) | β | -3.268^***^ (0.294) | β | 0.003 (0.001) |
|  | β*SD | -6.308 | β*SD | -6.314 | β*SD | 0.006 |
| USESCH | β | -7.536^***^ (0.779) | β | -7.59^***^ (0.710) | β | 0.014 (0.069) |
|  | β*SD | -6.225 | β*SD | -5.286 | β*SD | 0.939 |
| ENTUSE | β | -8.148^***^ (0.746) | β | -8.179^***^ (0.728) | β | 0.031 (0.042) |
|  | β*SD | -7.236 | β*SD | -7.263 | β*SD | 0.027 |
| INTICT | β | 9.955^***^ (0.661) | β | 9.954^***^ (0.661) | β | 0.010 (0.000) |
|  | β*SD | 9.308 | β*SD | 9.307 | β*SD | 0.001 |
| AUTICT | β | 23.529^***^ (0.775) | β | 23.533^***^ (0.774) | β | 0.004 (0.001) |
|  | β*SD | 21.076 | β*SD | 21.180 | β*SD | 0.104 |
| COMPICT | β | -2.931^***^ (0.796) | β | -2.934^***^ (0.794) | β | 0.003 (0.002) |
|  | β*SD | -2.597 | β*SD | -2.600 | β*SD | 0.003 |
| SOIAICT | β | -16.001^***^ (0.709) | β | -16.014^***^ (0.705) | β | 0.013 (0.004) |
|  | β*SD | -14.065 | β*SD | -14.076 | β*SD | 0.011 |
| ESCS | β | 47.930^***^ (0.663) | β | 47.920^***^ (0.660) | β | 0.010 (0.003) |
|  | β*SD | 39.398 | β*SD | 39.396 | β*SD | 0.002 |
| Gender (female = 0) | β | -28.506^***^ (1.039) | β | -28.488^***^ (1.035) | β | 0.018 (0.004) |
|  | β*SD | -14.253 | β*SD | -14.244 | β*SD | 0.009 |

Note. The coefficient of the regression model presented in this table is the mean coefficient of the 10 models. Heteroscedasticity-robust standard errors are listed in parentheses. The result of the model with HOMESCH (see Table 5) and that without HOMESCH (see Supplementary Table S3) were compared and no significant difference was found (see Supplementary Table S4).
