# Supplementary material for: Regression Analysis of ICT Impact Factors on Early Adolescents’ Reading Proficiency in Five High-Performing Countries
Source: Front Psychol. 2019 Jul 16;10:1646. doi: 10.3389/fpsyg.2019.01646 (PMC6646718; doi:10.3389/fpsyg.2019.01646)
Supplement: Supplementary file 5 [file Table_5.docx]

# Supplementary Table S5. The effect of ICT impact factors on reading proficiency without ENTUSE.

| Factor | Model 1  (PV1READ) | | Model 2  (PV2READ) | | Model 3  (PV3READ) | | Model 4  (PV4READ) | | Model 5  (PV5READ) | | Model 6  (PV6READ) | | Model 7  (PV7READ) | | Model 8  (PV8READ) | | Model 9  (PV9READ) | | Model 10  (PV10READ) | | Mean | |
| --- | --- | --- | --- | --- | --- | --- | --- | --- | --- | --- | --- | --- | --- | --- | --- | --- | --- | --- | --- | --- | --- | --- |
|  | β | β*SD | β | β*SD | β | β*SD | β | β*SD | β | β*SD | β | β*SD | β | β*SD | β | β*SD | β | β*SD | β | β*SD | β | β*SD |
| ***ICT availability*** | | | | | | | | | | | | | | | | | | | | | | |
| ICTHOME | -4.916***  0.361 | -8.052 | -4.593***  0.360 | -7.523 | -5.154***  0.356 | -8.442 | -4.353***  0.359 | -7.130 | -4.669***  0.366 | -7.648 | -4.305***  0.359 | -7.052 | -4.813***  0.362 | -7.884 | -4.765***  0.358 | -7.805 | -4.899***  0.360 | -8.025 | -5.279***  0.360 | -8.647 | -4.775***  0.360 | -7.821 |
| ICTSCH | -3.096  0.294 | -6.265 | -3.174***  0.296 | -6.132 | -2.830***  0.293 | -5.468 | -3.004***  0.297 | -5.804 | -2.925***  0.297 | -5.651 | -3.362***  0.292 | -6.495 | -3.411***  0.298 | -6.590 | -3.552***  0.294 | -6.862 | -2.902***  0.296 | -5.607 | -3.051***  0.299 | -5.895 | -3.131***  0.296 | -6.049 |
| ***ICT use*** | | | | | | | | | | | | | | | | | | | | | | |
| HOMESCH | -2.244***  0.692 | -2.114 | -2.274***  0.692 | -2.274 | -2.371***  0.681 | -2.234 | -1.916***  0.687 | -1.805 | -1.547***  0.695 | -1.457 | -1.942***  0.685 | -1.829 | -2.748***  0.689 | -2.589 | -0.883  0.695 | -0.832 | -3.085***  0.691 | -2.906 | -2.561***  0.698 | -2.413 | -8.179*** (0.728) | -7.263 |
| USESCH | -8.378***  0.783 | 5.946 | -8.940***  0.782 | -7.384 | -8.198***  0.766 | -6.772 | -9.068***  0.784 | -7.490 | -7.769***  0.785 | -6.417 | -8.772***  0.774 | -7.246 | -8.537***  0.777 | -7.052 | -9.026***  0.783 | -7.455 | -8.667***  0.777 | -7.159 | -8.985***  0.789 | -7.422 | -8.634***  0.780 | -7.132 |
| ***ICT attitudes*** | | | | | | | | | | | | | | | | | | | | | | |
| INTICT | 8.250***  0.651 | 9.183 | 7.956***  0.653 | 7.439 | 8.816***  0.647 | 8.243 | 8.276***  0.646 | 7.738 | 8.333***  0.650 | 7.791 | 8.670***  0.643 | 8.106 | 8.437***  0.653 | 7.889 | 9.043***  0.639 | 8.455 | 8.592***  0.652 | 8.034 | 8.752***  0.648 | 8.183 | 8.513***  0.648 | 8.359 |
| AUTICT | 22.075***  0.765 | 20.750 | 23.586***  0.774 | 21.227 | 22.033***  0.767 | 19.830 | 22.981***  0.769 | 20.683 | 22.618***  0.779 | 20.356 | 22.119***  0.766 | 19.907 | 22.431***  0.775 | 20.188 | 22.991***  0.773 | 20.692 | 22.341***  0.771 | 20.107 | 23.129***  0.777 | 20.816 | 22.630***  0.772 | 20.367 |
| COMPICT | -3.175***  0.791 | -2.546 | -3.281***  0.804 | -0.295 | -2.504***  0.791 | -0.225 | -3.502***  0.789 | -0.315 | -3.334***  0.8 | -0.300 | -2.676***  0.792 | -0.241 | -2.101***  0.800 | -0.189 | -4.274***  0.795 | -0.385 | -3.383***  0.795 | -0.304 | -3.847***  0.708 | -0.346 | -3.208***  0.787 | -0.289 |
| SOIAICT | -16.709***  0.705 | -13.794 | -17.232***  0.707 | -1.637 | -17.647***  0.698 | -1.676 | -17.070***  0.701 | -1.622 | -17.048***  0.712 | -1.620 | -16.665***  0.696 | -1.583 | -16.811***  0.708 | -1.597 | -17.042***  0.708 | -1.619 | -16.469***  0.707 | -1.565 | -16.676***  0.708 | -1.584 | -16.937***  0.705 | -1.609 |
| ***Student background*** | | | | | | | | | | | | | | | | | | | | | | |
| ESCS | 48.636***  (-0.653) | 39.627 | 47.786***  0.666 | 39.280 | 48.091***  0.660 | 39.190 | 48.483***  0.665 | 39.853 | 47.995***  0.673 | 39.452 | 48.146***  0.656 | 39.576 | 48.245***  0.665 | 39.657 | 48.269***  0.662 | 39.677 | 48.560***  0.66 | 39.916 | 48.988***  0.665 | 40.268 | 48.320***  0.664 | 39.719 |
| Gender (female=0) | -30.005***  -1.031 | -14.205 | -29.125***  1.032 | -14.563 | -28.943***  1.029 | -13.721 | -30.857***  1.030 | -15.429 | -30.245***  1.041 | -15.123 | -30.357***  1.024 | -15.179 | -32.030***  1.031 | -16.015 | -29.387***  1.028 | -14.694 | -30.077***  1.036 | -15.039 | -28.637***  1.037 | -14.319 | -29.966***  0.826 | -14.983 |

Notes: *N* = 37,155. The dependent variable is students’ readings score. Models 1 to 10 refer to the regression models for ten plausible values of the reading score. In PISA 2015, each student has 10 plausible values of reading scores (PV1READ~PV10READ). A higher plausible value reflects the higher reading proficiency. The regression model is estimated using Equation. Since the independent variables are derived based on IRT scaling, with one percent change of the independent variable, the dependent variable change by the coefficient multiplied by its standard deviation accordingly (β*SD). Heteroscedasticity-robust standard errors are listed in parentheses. Signif. codes: ****p* < 0.001, ***p* < 0.01, **p* < 0.05, *p* < 0.1. Nosignificant coefficients (0.1 < *p* < 1) are presented.
