# Supplementary material for: Regression Analysis of ICT Impact Factors on Early Adolescents’ Reading Proficiency in Five High-Performing Countries
Source: Front Psychol. 2019 Jul 16;10:1646. doi: 10.3389/fpsyg.2019.01646 (PMC6646718; doi:10.3389/fpsyg.2019.01646)
Supplement: Supplementary file 6 [file Table_6.docx]

# Supplementary Table S6. Comparison of the results of the regression models with and without ENTUSE.

| Factor | Regression model results with ENTUSE | | Regression model results without ENTUSE | | Differences | |
| --- | --- | --- | --- | --- | --- | --- |
| ICTHOME | β | -4.331^***^ (0.396) | β | -4.775^***^ (0.360) | β | 0.444 (0.036) |
|  | β*SD | -7.094 | β*SD | -7.821 | β*SD | 0.727 |
| ICTSCH | β | -3.265^***^ (0.295) | β | -3.131^***^ (0.296) | β | 0.134 (0.001) |
|  | β*SD | -6.308 | β*SD | -6.049 | β*SD | 0.259 |
| USESCH | β | -7.536^***^ (0.779) | β | -8.634^***^ (0.780) | β | 1.098 (0.001) |
|  | β*SD | -6.225 | β*SD | -7.132 | β*SD | 0.907 |
| HOMESCH | β | -0.325^***^ (0.700) | β | -8.179^***^ (0.728) | β | 7.854 (0.028) |
|  | β*SD | -7.235 | β*SD | -7.263 | β*SD | 0.028 |
| INTICT | β | 9.955^***^ (0.661) | β | 8.513^***^ (0.648) | β | 1.442 (0.013) |
|  | β*SD | 9.308 | β*SD | 8.359 | β*SD | 0.949 |
| AUTICT | β | 23.529^***^ (0.775) | β | 23.630^***^ (0.772) | β | 0.101 (0.003) |
|  | β*SD | 21.076 | β*SD | 20.367 | β*SD | 0.709 |
| COMPICT | β | -2.931^***^ (0.796) | β | -3.208^***^ (0.787) | β | 0.277 (0.009) |
|  | β*SD | -2.497 | β*SD | -1.559 | β*SD | 0.938 |
| SOIAICT | β | -16.001^***^ (0.709) | β | -16.937^***^ (0.705) | β | 0.936 (0.004) |
|  | β*SD | -14.065 | β*SD | -14.887 | β*SD | 0.822 |
| ESCS | β | 47.930^***^ (0.663) | β | 48.320^***^ (0.664) | β | 0.390 (0.001) |
|  | β*SD | 39.398 | β*SD | 39.719 | β*SD | 0.321 |
| Gender (female = 0) | β | -28.506^***^ (1.039) | β | -29.966^***^ (0.826) | β | 1.460 (0.213) |
|  | β*SD | -14.253 | β*SD | -14.983 | β*SD | 0.730 |

Note. The coefficient of the regression model presented in this table is the mean coefficient of the 10 models. Heteroscedasticity-robust standard errors are listed in parentheses. The result of the model with ENTUSE (see Table 5) and that without ENTUSE (see Supplementary Table S5) were compared and no significant difference was found (see Supplementary Table S6).
